# Human leukocyte Antigen-DM polymorphisms in autoimmune diseases

**DOI:** 10.1098/rsob.160165

**Published:** 2016-08-17

**Authors:** Miguel Alvaro-Benito, Eliot Morrison, Marek Wieczorek, Jana Sticht, Christian Freund

**Affiliations:** Protein Biochemistry Group, Institute for Chemistry and Biochemistry, Department of Biology, Chemistry and Pharmacy, Freie Universität Berlin, Berlin, Germany

**Keywords:** human leucocyte antigen-DM, polymorphism, major histocompatibility complex of class II, autoimmunity, peptidome, antigen presentation

## Abstract

Classical MHC class II (MHCII) proteins present peptides for CD4^+^ T-cell surveillance and are by far the most prominent risk factor for a number of autoimmune disorders. To date, many studies have shown that this link between particular MHCII alleles and disease depends on the MHCII's particular ability to bind and present certain peptides in specific physiological contexts. However, less attention has been paid to the non-classical MHCII molecule human leucocyte antigen-DM, which catalyses peptide exchange on classical MHCII proteins acting as a peptide editor. DM function impacts the presentation of both antigenic peptides in the periphery and key self-peptides during T-cell development in the thymus. In this way, DM activity directly influences the response to pathogens, as well as mechanisms of self-tolerance acquisition. While decreased DM editing of particular MHCII proteins has been proposed to be related to autoimmune disorders, no experimental evidence for different DM catalytic properties had been reported until recently. Biochemical and structural investigations, together with new animal models of loss of DM activity, have provided an attractive foundation for identifying different catalytic efficiencies for DM allotypes. Here, we revisit the current knowledge of DM function and discuss how DM function may impart autoimmunity at the organism level.

## 1. Antigen presentation and autoimmunity

The major histocompatibility complex (MHC), also known as human leucocyte antigen (HLA), stands out in genome-wide association studies as the most prominent genetic locus for a number of human autoimmune disorders [1,2]. The MHC locus also features high gene density, the highest degree of polymorphisms in the human genome, and a strong linkage disequilibrium (LD), which implies that genes are inherited as blocks rather than being shuffled in a segregated manner during recombination. Because most of the proteins encoded by the MHC locus are implicated in the function of the immune response, it has been difficult to assess single genes as specifically risk-conferring [3]. Thus, the link between MHC genes and autoimmunity has remained a complicated and poorly understood paradigm in a multifactorial disease context. Indeed, as many different factors (genetic and environmental) contribute to the aetiology of autoimmune disorders, it is probably more accurate to consider them as conditioning factors. Nevertheless, the specific contribution of MHC genes to the development of autoimmunity was first addressed in the 1970s. Studies by Schlosstein *et al*. [4] correlated HLA-B27, an MHC class I (MHCI) allele, and the autoimmune disorder ankylosing *spondylitis*. Since then, many other alleles from classical MHC class I (MHCI) and MHC class II (MHCII) genes have been linked to various autoimmune diseases [5].

Classical MHCI and MHCII proteins present peptides for T-cell surveillance, and are therefore essential for the initiation of cellular adaptive immune responses. T cells interact with the cognate peptide–MHC complexes via their T-cell receptor (TCR) and co-receptor molecules (CD4 or CD8). MHCI proteins present peptides to CD8^+^ T cells (cytotoxic T cells), resulting in cell lysis of the targeted cell, while MHCII molecules interact with CD4^+^ T cells (helper T cells) and lead to cytokine secretion and regulation of other immune cells, such as macrophages or B cells. There are important differences in the biological mechanisms of antigen processing and presentation by each MHC class [6], but for both classes it has been demonstrated that, while some alleles increase the susceptibility for certain autoimmune disorders, others seem to protect against disease. Interestingly, the majority of the risk-conferring alleles differ from the protective alleles by only a handful of residues, most of which are located in the peptide-binding groove or at the interaction surface with the TCR. This suggests a direct link between the biological function of MHC proteins—antigen presentation—and autoimmunity. Indeed, while autoimmune risk-conferring alleles seem to favour the presentation of particular peptides (targeted by the immune system in the particular disorder), the protective alleles seem to prevent their presentation. Wucherpfennig & Sethi [2] suggested that one of the key mechanisms linking MHC polymorphisms and disease is that certain MHC polymorphisms may allow or prevent antigen presentation of key self-peptides, depending on the physiological context. Namely, differences in antigen presentation between the thymus and the periphery are predicted to play a key role in the aetiology of many autoimmune disorders. Indeed, T-cell development (in the thymus)—especially the deletion of self-reactive T cells and the production of regulatory T cells (CD4^+^CD25^+^ regulatory T cells, T_regs_) that keep autoimmunity at bay—is one of the processes that clearly has a direct influence on autoimmune disease conditioning. In addition, the presentation of the key self-peptides in the target organ of the disease (periphery), where particular antigens are available at relatively high concentrations, must also be considered. Thus, it is likely that mechanisms involved in antigen presentation play a significant role in autoimmune conditioning [7].

In addition to the link between classical MHC proteins and disease, the relationship between other genetic factors related to antigen processing and presentation and autoimmune disease is becoming clearer. For example, certain polymorphisms of the transporter associated with antigen processing (TAP1 and TAP2) and the immunoproteasome subunits LMP2 and LMP7, both involved in MHCI antigen presentation, have been linked to type 1 diabetes (T1D) by genetic association studies [8]. Similarly, polymorphisms of ERAP1 (endoplasmic reticulum amino peptidase 1)—also involved in MHCI antigen processing and peptide loading—were linked by genetic association studies to ankylosing spondylitis [9]. More recently, it has been demonstrated that ERAP1 polymorphisms linked to disease impair the enzyme's peptidase activity [10,11]. For MHCII antigen presentation, the non-classical MHCII proteins HLA-DM and HLA-DO play accessory roles essential for antigen presentation. One of HLA-DM's primary functions is to act as a peptide editor for classical MHCII proteins, while DO acts as a negative modulator of DM activity in a restricted set of cell types. The combined activity of both proteins is essential for the appropriate functioning of MHCII antigen presentation, as reviewed below. Moreover, HLA-DM plays a major role in determining which peptides are preferentially presented, and to what extent they appear at the cell surface. We have recently demonstrated the reduced activity of a particular polymorphism of a *DMA* allele (DMA*0103) relative to the most abundant allele (DMA*0101), providing, for the first time, evidence for differential editing activity of natural variants of HLA-DM [12]. In this context, while genetic association studies have failed to make a clear connection between natural variants of DM and disease, reduced levels of DM activity in certain cellular compartments [13], modulation by other non-classical MHCII protein in murine models (HLA-DO [14]) and total loss of DM activity in an autoimmune-predisposing genetic background [15] have all been directly related to autoimmune conditioning. Difficulties in understanding the functional mechanism of HLA-DM, its low degree of polymorphism, and the complexity of the genetics of the MHC locus are likely to have hindered progress in investigating these topics, although the putative contribution of DM natural variants to autoimmune disorders has been touched upon by several reviews in the field [16–18].

### 1.1. Antigen presentation by MHCII molecules

**Figure 1.**
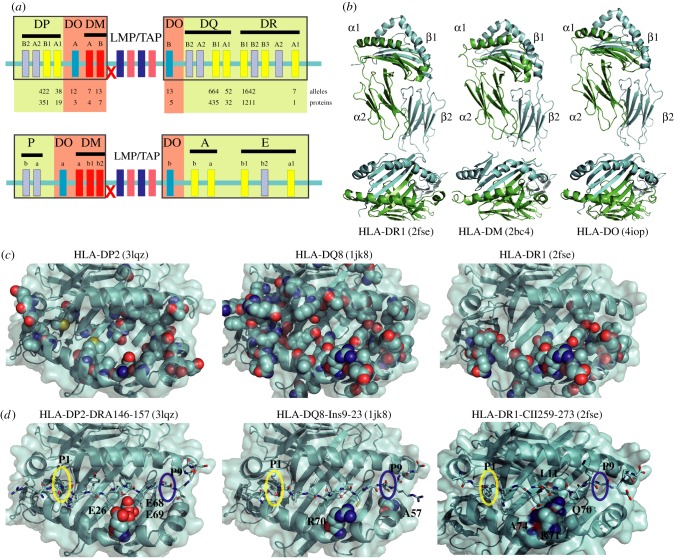
Genomic organization of the MHCII locus and the link between the MHCII structure and disease susceptibility. (*a*) Overview of the genetic organization of the MHCII locus in human and in mouse, and numbers of natural variants of the different MHCII proteins encoded by the MHCII locus in humans. Functional genes are shown as filled boxes and dashed light blue boxes represent pseudogenes which are not translated as functional proteins (e.g. *DPB2* and *DPA2* in humans and *I-Pa* and *I-Pb* in mouse). TAP and LMP genes belong to MHCI genes but are located within the MHCII locus. A yellow background indicates genes encoding classical MHCII proteins and a red background indicates the genes encoding for non-classical MHCII proteins. A red cross indicates a potential hotspot for recombination. (*b*) Example of three-dimensional fold of classical and non-classical MHCII proteins. The ectodomains are depicted in a cartoon representation and the classical MHCII protein HLA-DR1 (PDB: 2fse) and the non-classical MHCII proteins HLA-DM (PDB: 2bc4, bottom) and HLA-DO (PDB: 4iop, from which HLA-DM has been removed) are compared. Note that while the peptide has been removed from HLA-DR1, the non-classical MHCII proteins do not bind peptides. The ectodomains are shown with the alpha subunit in green and the beta subunit in cyan. The α1, β1 and α2 and β2 domains are indicated. The most important structural differences between classical and non-classical MHCII proteins are located in the α1 and β1 domains, and can be appreciated in the top view (bottom) (*c*) Structural details of MHCII proteins relevant for disease; polymorphic residues are depicted as spheres: HLA-DP (3lqz), HLA-DQ (1jk8) and HLA-DR (2fse). Peptides presented by these molecules and found in the pdb files have been removed to facilitate visualization. The epitopes are indicated in each structure. (*d*) The same proteins as in (*c*) are shown, and the polymorphisms associated with disease are represented as spheres. Peptides presented by these molecules are depicted in stick representations. The P1 and P9 pockets are highlighted with a yellow and a blue circle, respectively.

Classical MHCII proteins present peptides on the cell surface of professional antigen presenting cells (APCs) and in thymic epithelial cells to CD4^+^ T or developing T cells. In humans, classical MHCII proteins are encoded by genes mapping to the HLA class II locus, and include *HLA-DP*, *-DQ* and *-DR*; in mice, these are known as *I-P, I-A* and *I-E*, respectively (figure 1*a*). To date, several hundred allelic variants have been described for human classical MHCII molecules (figure 1*a*) [3]. Humans as well as mice do bear variable numbers of pseudogenes within the MHCII locus, though *DPB2/DPA2* and *I-Pa/IPb* are usually not transcribed into functional proteins. The non-classical MHCII proteins HLA-DM and HLA-DO are also encoded by genes mapping to the same genetic locus, and perform accessory functions required for antigen presentation. However, in contrast to classical MHCII proteins, HLA-DM and HLA-DO are considered oligomorphic (the number of alleles described are shown in figure 1*a*). All MHCII proteins, including the non-classical proteins, are heterodimers sharing a characteristic three-dimensional fold (figure 1*b*, upper panel), in which each subunit has: (i) a short cytoplasmic tail and a transmembrane domain; (ii) a membrane-proximal immunoglobulin domain (α2 and β2, respectively); and (iii) a membrane-distal domain contributing several β-strands and a discontinuous α-helix, which both become integrated into the peptide-binding domain of classical MHCII proteins when combined with the second subunit (α1 and β1).

In classical MHCII proteins, the peptide-binding groove (defined by the α1 and β1 domains, which fold into an eight-stranded twisted β-sheet, flanked by two contour-defining α-helices) determines what peptides are preferentially presented by each MHCII protein. Interestingly, the non-classical MHCII proteins HLA-DM and DO show important structural differences in this domain, accounting for their inability to bind peptides (figure 1*b*, lower panel). It is important to note that DO has not been crystalized as a single molecule, and the three-dimensional structure shown here has been taken from its complex with HLA-DM. Especially in the case of HLA-DM, the two α-helices are arranged almost in parallel, thereby closing the usually opened binding cleft. For classical MHCII proteins, the variability resulting from natural polymorphisms of the membrane-distal domains has been studied in detail [19] and, when mapped onto any of the available three-dimensional structures, can serve as a framework to explain the differences in binding properties of different MHCII allelic variants (figure 1*c*). Two critical features define the strength and specificity of a peptide towards a particular MHCII molecule: (i) hydrogen bonds between the peptide backbone and conserved residues of the MHCII helices and (ii) pockets defined by the surface of the protein (called P1–P9), which define the peptide's side-chains that result in the highest free energy of binding. The open edges of the binding groove of MHCII proteins allow the presentation of peptides of variable length (on average 13–25 amino acids), which will usually extend beyond the pockets that define the groove. In general, peptides bind to MHCII molecules in a defined orientation, with the N-terminal residues located close to the P1 pocket and the C-terminus close to the P9 pocket (figure 1*d*). Structural investigations have also shown that an inverted/non-canonical orientation is possible for the CLIP peptide (described below) [20,21]; however, the biological relevance of these findings has yet to be addressed *in vivo*.

Classical MHCII genes are co-translationally translocated into the ER where they fold with the assistance of the invariant chain protein (Ii or CD74), building up a nonameric complex (Ii_3_MHCII_3_), which traffics through the Golgi and reaches late endosomal compartments [22], also referred to as MHCII-like compartments (MIICs). In MIICs, most of the Ii chain is degraded by specialized proteases [23,24], and only a short nested set of peptides known as the class II associated invariant chain peptide (CLIP) remains bound to the MHCII molecules. This ‘placeholder’ peptide prevents premature association of polypeptides to MHCII molecules in the ER as demonstrated by Busch *et al*. [25] using HeLa transfectants, and facilitates the efficient interaction of active MHCII proteins with antigens in the acidic milieu of MIICs [23,26]. Many routes of antigenic uptake converge into these MIICs [27], which contain functional MHCII proteins, proteases (mainly cathepsins) and the accessory molecules (HLA-DM and -DO) required for efficient peptide exchange of MHCII molecules. In these specialized vesicles, concomitant antigen degradation and antigenic peptide exchange facilitated by DM result in the formation of stable peptide–MHCII complexes, which then traffic to the cell surface; however, only those that are kinetically stable over the range of the approximately 3 pH-unit difference experienced when travelling from the late endosomes to the cell surface are finally presented to CD4^+^ T cells. It is worth noting that the specific set of active proteases, antigen availability and the presence or the absence of accessory molecules in the particular physico-chemical environment of MIICs will all contribute to defining the antigenic complex ultimately formed and presented. Indeed, alternative routes for antigen processing and loading, or even cross-presentation between MHCI and MHCII routes, have been described as having an impact on the peptides presented by MHCII molecules [28]. A recent study by Clement *et al*. [29] based on a DR1 transgenic mouse and pooled material from human patients showed that the lymph dendritic cell (DC) peptidome includes antigenic peptides from a number of sources, including epitopes which have not undergone DM editing. Eisenlohr and co-workers [30] have already proposed that MHC antigen presentation should be seen as a network of antigen processing routes rather than limited to the classical pathway. Thus, the peptidome presented by classical MHCII proteins will be the result of the conventional processing pathway described here, as well as alternative routes reviewed elsewhere [27,29]. Moreover, it is important to note that for each type of APC, there are particular features defining expression of classical and non-classical MHCII proteins, specific proteolytic activities and preferential routes for antigen processing. Thus, these differences will most probably impose specific constraints onto the mechanisms of antigen processing and presentation for each specific cell type.

### 1.2. Established links between MHCII polymorphisms and autoimmunity

Many previous studies have described the link between classical MHCII polymorphisms and autoimmune diseases. In table 1, we summarize some of the well-characterized human polymorphisms contributing to rheumatoid arthritis (RA) and T1D. As recently reviewed by Tsai & Santamaria [36], there are hotspots in MHCII molecules which seem to favour the binding of particular self-peptides that will ultimately be recognized by self-reactive TCRs. In the case of the link between DR4 molecules and RA, it seems that single-nucleotide polymorphisms (SNPs) resulting in polymorphisms in DRβ71 and DRβ74 favour binding of collagen II-derived peptides (CII). As may be expected, DR1 alleles sharing DRβ71 residues are also linked to RA (figure 1*d*). Similarly, in the case of T1D, it seems that the presence of an amino acid other than β57Asp, β57Ala in DQ8 alleles (figure 1*d*), favours the binding of insulin-derived peptides (the same applies for the murine I-A^g7^ allele). Although polymorphisms of DP alleles have received less attention, recent studies have revealed that three different Glu residues in positions DPβ26, DPβ68 and DPβ89 are related to a particular beryllium-induced autoimmune disease [37,38] (figure 1*d*). Indeed, two other common features of classical MHCII proteins associated with an elevated risk for autoimmunity are low affinity for CLIP and low DM susceptibility [16]. However, these biochemical features of classical MHCII risk-conferring alleles do not solely explain the existence of self-reactive CD4^+^ T cells. Thus, besides taking into account what is known about the contribution of MHCII polymorphisms to the loss of tolerance to self, it is essential to understand the contribution of other key elements involved in antigen processing and presentation.

**Table 1.** Examples of MHCII-mediated autoimmune disorders and model self-antigens.

disease	MHCII	key polymorphism(s)	self-antigen	refs
rheumatoid arthritis	DRB1*0101 DRB1*0401	β11 (Leu/Val) β70 (Arg/Gln) β71 (Lys) β74 (Ala)	collagen II	[31]
type 1 diabetes	DRB1*0401 DRB1*0405	β57 (Asp/Ser) β71 (Lys/Arg) β74 (Ala) β86 (Gly)	insulin GAD65	[32,33]
	DQA1*0301 DQB1*0302	β57 Not Asp (Ala)	insulin GAD65	[34,35]

Autoreactive T cells (CD4^+^ or CD8^+^) are normally eliminated at the early stages of their development in the thymus by the so-called central tolerance mechanisms, which ensure that TCRs generated upon somatic recombination are able to interact with self-MHC proteins (positive selection), and that potential self-reactive TCRs are eliminated (negative selection). Both selection processes take place in the thymus, and both are driven by differentiation and/or apoptotic/anergic signals that the developing thymocytes receive upon TCR–peptide–MHCII complex interaction [39]. The current affinity model for TCR selection states that in the case of positive selection, survival signals are generated from a strong interaction of the TCR with MHCII molecules in the thymic cortex, while high-affinity interactions of TCRs with a peptide–MHCII complex in the thymic medulla drive developing T cells to apoptosis during negative selection [39,40]. This model implies that failures in positive selection result in non-functional TCRs, and that, in the case of failed negative selection, there will be a higher likelihood of release of self-reactive T cells to the periphery. Functional negative selection seems to be relatively inefficient, as, for example, in the case of multiple sclerosis (MS) CD4^+^ T cells bearing self-reactive TCRs associated with MS have been found in diseased and healthy individuals [41]. Recent investigations have emphasized the role of T_regs_ on constraining self-reactivity [42]. T_reg_ cells (CD4^+^CD25^+^) are mostly selected in the thymic medulla, but conventional CD4^+^ T cells can also differentiate into T_regs_ in the periphery [43]. Although this particular T-cell subset can be induced by peptide–MHCII nanoparticles and efficiently restrict self-reactivity [44], long-lasting and effective protection seems to be driven by negative selection and T_reg_ differentiation in the thymus [42]. From the perspective of antigen processing and presentation, both negative selection mechanisms and T_reg_ differentiation in the thymus are based mostly on the expression and presentation by thymic epithelial cells of the so-called tissue-restricted antigens, which are facilitated by the protein autoimmune regulatory element. Additionally, migratory DCs [45] and B cells [46] have also been described to be active during negative selection processes. A comprehensive review on T-cell tolerance acquisition has been recently published by Perry & Hsieh [47]. To date, there exists experimental evidence for the contribution of classical MHCII molecules to the loss of self-tolerance at three different levels.

#### 1.2.1. Differences in processing conditions or available antigens, leading to differences between the peptidomes presented in the thymus and in the periphery [7]

In this way, substantial differences in the antigens presented during thymic selection and those encountered in the periphery are generated. One example is when a pathogenic peptide is not present in the sequence of the isoform expressed in the thymus. This is the case for the proteo-lipid-protein implicated in MS, for which self-reactive T cells are not deleted [48]. Post-translational modifications taking place exclusively or primarily in the periphery can also modify peptides in such a way that they behave differently when binding to MHCII molecules or in their recognition by TCRs (as peptide–MHCII complexes) [49]. In some instances, low affinity of a particular peptide for MHCII proteins also seems to be important for the release of self-reactive T cells. Low affinity would be likely to result in low or abolished presentation of the antigenic peptide during the acquisition of tolerance if the antigen concentration is limiting, as proposed for a cryptic epitope of MBP, also related to MS [50]. All of the above examples are specific for particular antigens. Moreover, recent attempts to understand the human thymic peptidome in a broader sense have demonstrated that negative selection is a dynamic process. Thus, the matrix of peptides presented by MHCII is very broad and changes in an age-dependent manner [51–53]. Although differences in the presented peptidome in thymus versus peripheral tissues could explain the escape of autoreactive T cells to the periphery, it seems difficult to experimentally address these differences in a systematic manner.

#### 1.2.2. Inefficient T-cell deletion during negative selection resulting from atypical peptide–MHCII–TCR interactions [54]

The specific features of self-reactive TCRs, with regard to the interaction with peptide–MHCII complexes, enable them to escape deletion mechanisms. Thus, for instance, the self-reactive TCRs Ob1A12 and Hy.1B11 seem to dock into the MBP-DR2 and MBP-DQ1 cognate complexes in an unconventional manner [55,56], which allows them to escape negative selection. While the interaction between peptide and MHCII is usually of high affinity, these TCRs have generally low affinity for the peptide–MHCII complex. Besides, the different geometry adopted by the TCR complex shall result in the repositioning of the co-receptor molecule CD4. Consequently, the assembly of the intracellular signalling complex formed upon TCR–peptide–MHCII engagement might also be altered. It has been suggested that together with low affinity of the TCR for the peptide–MHCII, the repositioning of CD4 might result also in different phosphorylation patterns and possibly T-cell activation thresholds in self-reactive T cells [54].

#### 1.2.3. Inefficient T_reg_ development in the thymus and/or in the periphery

Tsai & Santamaria [36] have proposed that the hotspots described for MHCII risk alleles that lead to failures in central tolerance are also involved in differences in T_reg_ selection. According to this model, in some cases, the polymorphism encoding for a risk allele results in a lower number of T_reg_ cells generated. This hypothesis has come to light after intensive research using combinations of protective versus risk-conferring murine backgrounds expressing specific self-reactive TCRs. Additionally, T_reg_ development (in the thymus) or induction (in the periphery) depends on, among other factors, the presentation of self-peptides [57,58]. As recently shown by two groups, non-classical MHCII proteins, especially HLA-DM, play an important role in these processes [14,15].

Considering the multifactorial aetiology and the complexity of autoimmune disorders, it is likely that no single mechanism described above will lead to the complete loss of tolerance to self. Nevertheless, it is evident that antigen presentation plays a major role during tolerance acquisition, regulation of self-reactivity, and in the targeting mechanisms that result in self-reactivity in the periphery. The central contribution of HLA-DM on editing of the peptidomes associated with MHCII proteins makes it likely that the system will be especially sensitive to any deviation from the ‘normal’ activity of this protein. Differences in activity could then have drastic consequences, considering that different antigen processing conditions are expected between the thymus and the peripheral tissues [2].

## 2. The central role of HLA-DM in MHCII antigen presentation

HLA-DM is a heterodimeric non-classical MHCII molecule whose expression is mostly restricted to professional APCs. Unlike classical MHCII molecules, DM does not bind peptides, and its function was initially described as the removal of the invariant chain-derived peptides (CLIP) in late endosomes, thus allowing for binding of antigenic peptides to MHCII proteins [59–61]. During the last 20 years, a number of research groups have described more general functions for DM as a peptide editor [62–64] and as a chaperone of empty MHCII proteins [65,66]. These two functions, and their importance to adaptive immunity, are reviewed by Sant *et al.* [67] and Busch *et al*. [68], and are discussed in the following sections. Essentially, HLA-DM selects for kinetically stable peptide–MHCII complexes and chaperones empty MHCII proteins, protecting them from aggregation. Especially relevant to the current discussion, kinetic stability seems to be related to immunogenicity [63], and frequently complexes of MHCII molecules with peptides that are able to elicit strong immune responses (immunodominant epitopes) are formed in the presence of DM. By contrast, cryptic epitopes are usually kinetically unstable, and are only able to elicit immune responses when supplied directly as peptides or unfolded proteins [69]. Differences in antigen processing, and especially in DM editing function (as summarized below), are proposed to be key factors for the presentation of cryptic epitopes, which are suspected to play an important role in the pathogenesis of autoimmune disorders [69]. Moreover, the consequences of total loss of DM activity in particular murine models indicate a certain correlation with the inability to fight infections and autoimmune disease, as discussed in the following sections.

### 2.1. HLA-DM genes, expression and regulation

**Figure 2.**
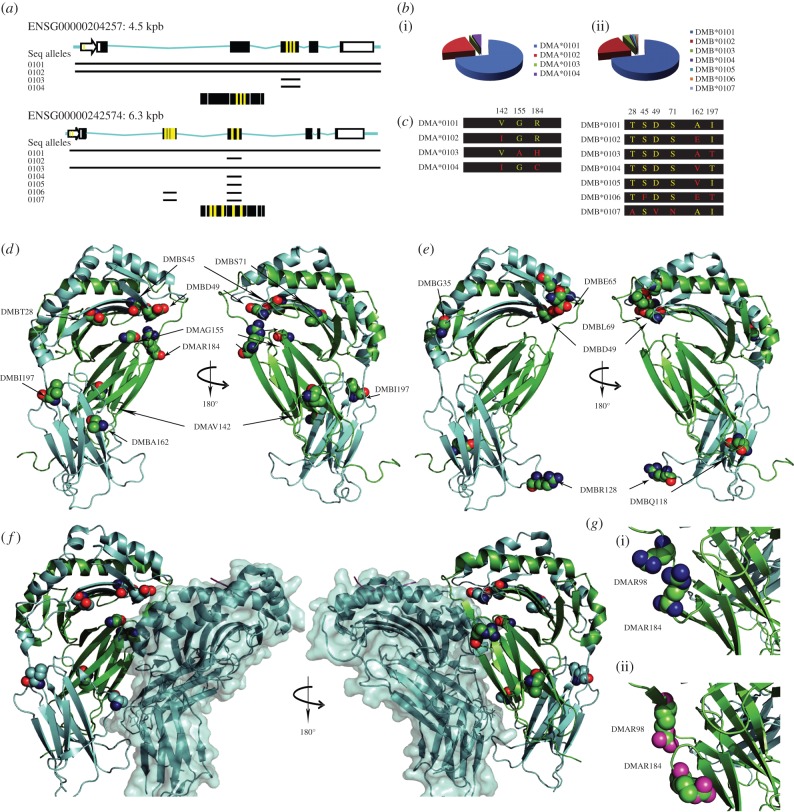
HLA-DM genes and proteins. (*a*) Structure for the *HLA-DMA* and *HLA-DMB* genes. ENSEMBL accession numbers are provided as well as the gene size in kbp. Exons are shown in boxes, in which the polymorphic regions are highlighted in yellow. Black lines show sequenced regions in different alleles. (*b*) *HLA-DMA* (i) and *DMB* (ii) allelic frequencies shown as pie charts. (*c*). Protein chains for *DMA* and *DMB* allelic variants are shown with the polymorphic residues highlighted. The most abundant and characterized *DMA* and *DMB* alleles (DMA*0101 and DMB*0101, respectively) are shown on top, and the polymorphic residues are shown in yellow. Any change on the amino acid level is shown in red. (*d*) Cartoon representation of the three-dimensional structure of HLA-DM showing the polymorphic residues as spheres. (*e*) Three-dimensional structure of HLA-DM (PDB: 2bc4), where residues identified in other studies as affecting enzymatic activity in B-cell lines are shown as spheres. (*f*) Positioning of the polymorphic residues of HLA-DM in the context of the HLA-DM–HLA-DR1 three-dimensional structure (PDB: 4fqx). (*g*) Zoom-in of the polymorphic residue DMα184R showing the structural rearrangement between the HLA-DM-free (i) and in complex with HLA-DR1 (ii). The three-dimensional structures are shown as cartoons and the DMαR184 (variable residue in *DMA* alleles) and the DMαR98 (important for the DM–DR interaction *in vitro*) residues are shown as spheres.

*HLA-DMA* and -*DMB* genes map to the MHCII locus and are located between the classical *HLA-DP* and *-DQ* MHCII genes, which themselves are flanked on the 5′ side by *HLA-DOB* and on the 3′ side by *LMP–TAP* genes (figure 1*a*) [70]. *DMA* and *DMB* display considerably lower degrees of polymorphism when compared with the overall MHCII locus, and especially when compared with classical MHCII proteins. To date, only seven allelic variants of *DMA*, resulting in only four different proteins, and 11 of *DMB*, resulting in seven proteins, have been described [71]. Of these 18 allelic variants, the complete coding DNA sequence (CDS) has only been sequenced for DMA*0101, DMA*0102, DMB*0101 and DMB*0103; for all other variants, sequencing was restricted to only certain exons (figure 2*a*). The 1000 Genomes Project (www.1000genomes.org) has nevertheless identified a high number of SNPs and other genetic variants with low frequencies, while also confirming the sequence of the allelic variants which have already been described.

In humans, each individual carries at least two *DMA* and at least two *DMB* alleles. *DMA* and *DMB* alleles are unevenly distributed throughout the studied populations. While DMA*0101 and DMB*0101 alleles are present in more than 95% of the studied subjects and DMA*0102 and DMB*0102 are represented in 30% of the populations investigated, the other allelic variants of *DMA* and *DMB* are found in less than 10% of the studied individuals (data extracted from www.allelefrequencies.net; figure 2*b*). Because of the intrinsic LD within the HLA locus, the presence of particular *DMA*–*DMB* haplotypes can be expected [72]. However, the occurrence of haplotypes for the HLA locus has been mostly restricted to genes encoding for classical MHC proteins [3,73], and only a handful of the studies discussed in §3 have addressed this issue for HLA-DM. Considering the heterodimeric nature of DM and that its activity is dependent on both subunits, it is crucial to understand the degree to which specific DM allotypes are present, in order to appropriately identify and estimate their contribution to any disease.

Because the use of murine models has facilitated a large part of the research that has led to our fundamental knowledge of DM function, it is important to note that DM genes in this organism show a unique organization relative to other mammals. H2-DM, the murine homologue of HLA-DM, is encoded by three different genes: *H2-DMa* (homologue to *DMA*), and *H2-DMb1* and *H2-DMb2*, which are both homologues of *DMB* [74] (figure 1*a*, bottom panel). Interestingly, both *H2-DMb* genes seem to be derived from a recent gene duplication process that occurred exclusively in mice, as they are not present even in taxonomically related organisms such as rats [75]. Both of the encoded heterodimers are functional [76]. As in humans, restricted polymorphism for H2-DM has been described and, interestingly, most of the variability lies in the genes encoding the beta subunits, while the *H2-DMa* gene is considered to be oligomorphic [77,78]. Despite the above-mentioned differences, the *DMA* and *DMB* genes share the same gene intronic–exonic arrangement in mice and humans, and the protein regions displaying variability in the alleles described for DM are spread across the entire protein sequence (figure 2*c*,*d*).

HLA-DM expression, as for most of the MHCII genes, is controlled by the regulatory factor X1 (RFX1) and the class II trans activator (CIITA). RXF1 binds specific sites found in the promoter regions (X-boxes) of MHCII genes, and acts to nucleate binding of the enhanceosome, which then recruits CIITA, finally resulting in transcription. B-cell lines defective for CIITA show basal levels of all MHCII proteins except DO and DRA, indicating that only these MHCII proteins are strictly dependent on CIITA [79]. However, a feature shared by all MHCII genes is that they are upregulated by IFN-γ, and this has been correlated to the induction of CIITA expression [80]. The lack of expression of MHC genes, linked to defects of RXF1, CIITA or other transcription factors, is known as bare lymphocyte syndrome (BLS), and results in severely immunocompromised individuals [80]. The above-mentioned master regulation for MHC gene expression by RFX1 and CIITA makes defects in the expression of particular MHC proteins unlikely. However, downregulation of DM expression resulting in lower protein levels has been detected in RA patients [81]; this is discussed in more detail below.

### 2.2. Biochemical and structural insights into the function of HLA-DM

HLA-DM functions as a peptide exchange catalyst and, to date, there is no evidence that it binds peptides itself. The peptide loading and exchange mechanism catalysed by DM is an enzymatic reaction and has long remained elusive due to the transient nature of the DM–MHCII interaction. Recent biophysical and structural studies, however, have shed light on the general mechanism of DM's peptide-exchange catalysis [82–84], as well as that of DM inhibition by DO [85,86]. These seminal papers by the Wucherpfennig, Stern and Mellins laboratories, combining crystallographic and *in vitro* functional studies (peptide exchange catalysis), allow us to better understand important intramolecular interactions between DM–DR and DM–DO that could be affected in natural variants of these proteins. In the reported DM/DR1 and DM/DO crystal structures, the interaction surface is mostly dominated by the alpha subunits of both molecules, with smaller but considerable contribution by the beta subunits. Previous reports, primarily from the Mellins laboratory [87,88], helped to establish the lateral interaction between DM and DR, and also revealed that residues distant from the interaction site in the three-dimensional structure could impact DM function (figure 2*e*). Interestingly, some of the *DMA* and *DMB* polymorphisms map closely to this interface, and may affect DM function (figure 2*d*,*f*) by directly affecting DM catalytic efficiency, or its inhibition by DO.

As a catalyst, DM does not exert the same efficiency on every peptide–MHCII complex. Thus, even for a single allelic variant, there are peptide–MHCII complexes that are more prone to be edited by DM than others (DM-susceptible complexes). The structural determinants of MHCII complexes with regard to DM activity have long remained a matter of debate [59,63,83,84,89–98]. It seems that a major contribution of the P1 pocket, together with the overall dynamics of the whole peptide-binding groove, define this interaction [83,84,97]. The structure and dynamics of the P1 pocket seem to be of relevance, as MHCII alleles with low DM-susceptibility are predicted to display variations in these features, and have been linked to autoimmunity. For example, for the HLA-DQ2 allelic variant DQA1*0502 [98–100], poor DM editing is related to a deletion of Ser53 in a region directly involved in DM binding. Differential editing functions of DM polymorphic variants have also been predicted for peptides bound in an unusual manner to MHCII. Thus, using *in vitro* studies, several groups have found that an individual peptide can bind in different registers [101] or orientations [20,21], and that DM is observed to accelerate the formation of a thermodynamically stable complex. Interestingly, the formation of different peptide–MHCII isomers has been associated with the activation of different sets of CD4^+^ T cells involved in autoimmunity; these links are discussed in the following section. Whether DM heterodimers bearing different polymorphisms will modulate these outcomes is an important, yet still challenging, question.

*In vitro* studies with DM mutants addressing the role of small molecules modulating the activity of DM [102] revealed that the polymorphic residue DMβD31, in combination with other mutants, may result in more efficient catalytic activity (figure 2*e*). However, to date, the recent study from our group is the only published work that has directly addressed how DM peptide exchange activity is affected by naturally occurring polymorphisms [12]. Our study was restricted to the analysis of a low-abundance polymorphic variant of *DMA*, DMA*0103, which was compared to the most abundant variant DMA*0101 [12]. Our studies revealed that DMA*0103-containing heterodimers showed a reduced catalytic efficiency in peptide exchange assays when using different DR molecules. Moreover, we identified the DMαG155A substitution as contributing most to the observed effect. Interestingly, the second polymorphic residue found in the DMA*0103 allele, DMαR184H, seems to mediate a partial recovery of the negative effect imposed by the DMαG155A mutation. Interestingly, this residue (DMαArg184) is located in one of the few regions of DM where substantial structural rearrangements take place upon engagement of DR, including the movement of the DMαArg98 lateral chain, which has been shown to be important for the DM–DR interaction [81] (figure 2*g*). Moreover, in the DMA*0104 allele, the SNP found would lead to a missense mutation, resulting in a cysteine residue at position 184. It is difficult to predict the effect of any substitution in the DM–DR or DM–DO interactions, and therefore the consequences, if any, on DM activity for any allelic variant. Two different studies from the laboratories of Mellins and Stern addressing the DM–DO interaction also investigated the relevance of DM polymorphisms in peptide exchange activity. The substitutions DMβA144 V and DMβA144E, representing DMB*0102 and DMB*0105, respectively, were introduced into DM heterodimers and tested for interaction strength with DO by FRET, and for peptide exchange activity in endpoint ELISA experiments using DR4 in complex with CLIP. In this case, the authors detected only little or no effect of mutations on DM function [85,86].

### 2.3. Investigations of HLA-DM function in a cellular context and the impact of HLA-DM in the selection of immunodominant epitopes

**Figure 3.**
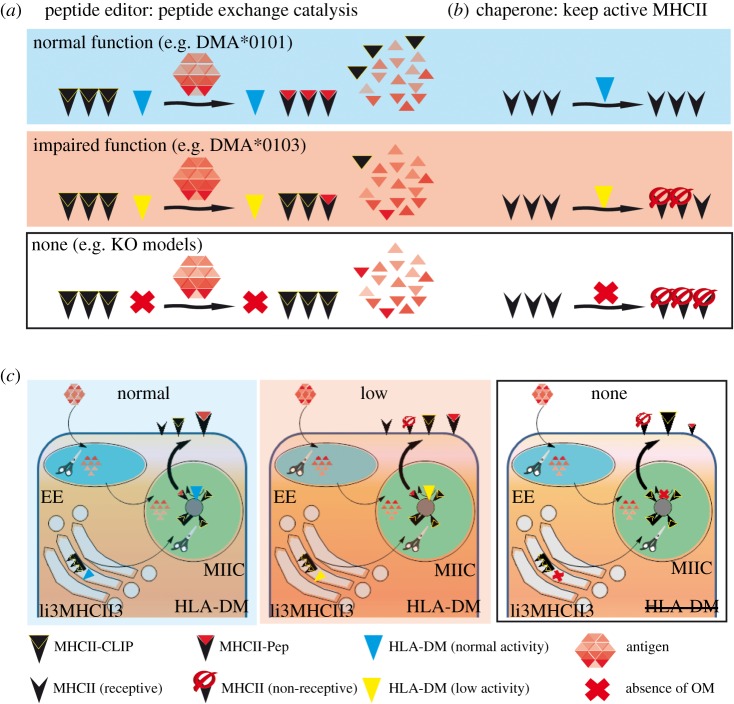
Known cellular functions of HLA-DM and consequences of total or partial loss of DM function. MHCII molecules are shown as black ‘V's. CLIP peptides are shown as black triangles. Antigenic peptides are shown as red triangles (lightness represents the affinity for the MHCII, with dark representing high affinity). HLA-DM is shown as a large triangle, and the colour correlates with its catalytic activity (cyan: normal; yellow: impaired) and in the case of total loss of activity a red ‘X’ is shown. (*a*) Peptide editor function of HLA-DM. DM function leads to the formation of highly stable peptide–MHCII complexes. MHCII molecules in complex with CLIP encounter antigenic peptides of different affinities. Under conditions of normal HLA-DM activity (yellow triangle, upper panel), CLIP is exchanged by higher affinity antigenic peptides resulting in stable peptide MHCII complexes. In the case of no HLA-DM activity, CLIP will mostly remain associated with MHCII molecules (lower panel). In the case of HLA-DM impaired activity not all CLIP will be dissociated from MHCII proteins. The arrows represent the relevant antigen processing conditions for each antigen (unfolding and/or proteolysis). (*b*) Chaperone function of HLA-DM. The chaperone function of DM rescues empty MHCII molecules from degradation. In the absence of HLA-DM activity, MHCII molecules with low affinity for CLIP collapse and are unable to present peptides. In conditions of normal HLA-DM activity, a large pool of the empty MHCII molecules will be rescued (upper panel). In the total absence of HLA-DM function (lower panel), the pool of rescued MHCII proteins will be lower (and therefore the total levels of MHCII proteins will also be lower). Impaired catalytic HLA-DM activity would lead to an intermediate situation. (*c*) HLA-DM activity in a cellular context and its impact at the peptidome level. HLA-DM and MHCII proteins (as nonameric complexes Ii3MHCII3) assemble in the ER and traffic through the Golgi. HLA-DM favours peptide exchange and acts as a chaperone mostly in MIIC (late endosomal compartments/MHCII compartments). HLA-DM is supposed to be more effective in MIIC compartments where antigens, mostly internalized, are degraded by cellular proteases (represented as scissors). The peptidome associated with MHCII proteins is represented with the same symbols as in (*a,b*), and the sizes indicate the relative amount of each complex. Normal DM expression levels result in a concrete peptidome (left, mostly composed by high affinity antigenic peptides) which is substantially altered in the absence of DM activity (right, where CLIP is the most abundant peptide and the presence of non-receptive MHCII molecules is higher). A partial loss of HLA-DM activity (due to catalytic impairment, centre) is expected to have consequences on the MHCII-associated peptidomes.

The role of HLA-DM in antigen processing was first studied in mutant B-cell lines by Mellins and collaborators [60,89,103]. Similarly, using a BxT cell hybrid depleted of all MHCII genes (T2), the Cresswell laboratory was able to use DM to restore the normal antigen processing and presenting ability of this cell line [104]. These papers pinpointed the central role of DM as a peptide-exchange catalyst (figure 3*a*) and facilitated the study of its cellular function. Later studies using the T2 cell line revealed DM's additional ability to chaperone MHCII proteins (figure 3*b*) [65]. After synthesis, both HLA-DM chains are translocated into the ER lumen, where they assemble. Then, DM traffics through the Golgi, finally reaching the endosomal compartments with the assistance of a localization motif (YPTL) present on the cytoplasmic tail of its beta subunit [105,106]. DM has been demonstrated to interact with Ii during cellular transport, but its distribution and folding does not seem to be affected by this protein [106,107]; indeed, the relevance of this interaction has not yet been addressed.

DM is widely distributed in cells, and its activity could contribute to peptide editing in many different subcellular compartments (figure 3*c* shows its major contribution on MIIC compartments) [108–110]. Expression levels of DM protein detected in cells are around one-fifth that of HLA-DR, and are estimated to be at a 1 : 20 molar ratio of DM : total MHCII [111]. It is also important to note that the DM peptide exchange catalytic mechanism is pH-dependent [89,102,112], and therefore its contribution to peptide editing may differ at different subcellular locations [113]. For this reason, its activity at the cell surface was initially disregarded [62,110], although later reconsidered [114]. One of the reasons argued for a low DM contribution to peptide exchange in different compartments, especially in the cell surface, was the pH-dependency for DM–MHCII interaction *in vitro* [84]. However, the Neefjes laboratory demonstrated at the cellular level by Försters resonance energy transfer (FRET) that DM–DR interaction is pH-independent [115]. Moreover, Thibodeau *et al*. have also shown that DM indeed can catalyse peptide-exchange on the cell surface modulating T-cell responses [116]. It is worth noting that those studies were based on overexpression of the different MHC proteins under consideration and the physiological barriers to interaction may be overcome at high expression levels.

The interaction of DM and DR was investigated in detail using B-cell lines by two studies of the Mellins laboratory [87,88]. While the first study generated mutations of the DR molecule itself, the second focused on mutations of both *DMA* and *DMB*. These studies included the generation of mutations spatially proximal to the DM–DR interface as it is now known [84], and show dramatic effects on DM activity, as detected by CLIP accumulation at the cell surface. Moreover, and in line with the biochemical studies described in the previous section, it is generally accepted that allelic variants of MHCII proteins are differentially affected by DM activity. Thus, as an interesting and illustrative example, for different DR4 alleles it has been shown that those with low CLIP affinity are unable to form highly stable peptide–MHCII complexes in the absence of DM (e.g. DRB1*0401 and DRB1*0404 [117]), and that the chaperone function of DM is able to overcome the lack of Ii and enhance their expression levels [66,118]. Moreover, in the case of DRB1*0401 (which has been linked to RA), it was shown that there are important changes to the composition of the presented peptidome of cells expressing this particular allele in the presence and absence of DM, with a clear shift towards peptides with higher affinities and different peptide sources when DM is present [119]. The requirements for DQ and DP to form stabilized complexes in the presence of DM are more variable, and generally less well understood than for DR [100,120]. For DQ alleles, a few experimental findings have been documented, and include the above-mentioned study of DQ2 [99,100], another study addressing DQ5 [120], and a recent study by Jensen's group which has focused in DQ alleles related to autoimmune disorders [98]. It seems that DQ2 and DQ8 alleles are relatively unaffected by DM, and for DQ5 there is no requirement for DM to form stable complexes, although DM does increase their formation [120]. In the study by van Lith *et al.* [120], the alleles DPB1*040101 and DPB1*1701 (both in combination with (DPA1*010301)) were also investigated, and the conclusion reached was that DP requires neither Ii nor DM to form stable complexes. It is important to note that most of these studies have correlated DM's function to the ability of classical MHCII molecules to form SDS-stable heterodimers (protein complexes that migrate in an SDS-PAGE as stable dimers). SDS-stability of peptide–MHCII complexes generally correlates well with the stability of a given peptide–MHCII complex in solution, but true kinetic stability specifically refers to a measurable thermodynamic parameter and should not be confused.

The first experiments addressing loss of DM function in a cellular context revealed that in its absence, besides CLIP accumulation at the cell surface, restricted T-cell hybridomas were poorly or not at all activated [60,104]. Later studies elucidated the role DM plays in regulating the presentation of immunodominant epitopes derived from extracellular antigens; these studies are reviewed by Sant *et al*. [67] and Busch *et al*. [68]. Essentially, DM favours the selection of immunodominant (kinetically stable) over cryptic (low stability) epitopes, which seems to be important for the presentation of pathogenic peptides in autoimmunity [69]. Thus, in the context of autoimmunity, the presentation of DR4-restricted epitopes from GAD65 [121], which has been linked to T1D, is inversely proportional to the relative amounts of DM expressed by the APCs. Similarly, Amria *et al*. [122] demonstrated that the relative activity of DM in B cells and macrophages negatively regulates the presentation of pathogenic epitopes of CII-derived peptides to restricted T-cell hybridomas. By contrast, RA-related epitopes derived from HCgp39 were shown to require DM for effective antigen presentation [123]. DM editing was associated with the formation of different isomers of peptide–MHCII that activate different CD4^+^ T-cell populations, and was suspected to be related to self-reactivity [124,125]. These studies suggested that antigen processing of the whole proteins leads to the activation of type A CD4^+^ T cells, while antigens administered as peptides led to the activation of another subset of CD4^+^ T cells, called type B. The preferential formation of one or the other isomer, and hence the activation of the CD4^+^ T cells, was related to low or absent DM editing function. Peptide isomerism and activation of type B CD4^+^ T cells has been directly related to autoimmunity in the case of murine I-A^g7^ in complex with the InsB9-23 peptide [13,126,127]. Moreover, in the review by Mohan & Unanue [128], the absence of DM function is argued as one of the elements allowing the presentation of unconventional isomers activating self-reactive T cells. The requirement of HLA-DM for the presentation of antigenic epitopes, however, is not completely understood, and receptor-mediated endocytosis has even been shown to be sufficient for the presentation of immunodominant peptides in cells lacking DM [129]. Thus, DM editing seems to be important editing seems to be important for the elimination of self-antigenic peptides that activate self-reactive CD4^+^ T cells. Consequently, impaired function of DM due to natural variation could result in low editing activity, increasing the presentation of cryptic epitopes related to autoimmunity.

DM's interaction with DO was first observed in cells [130], and *in vitro* studies were used to assess its regulatory role on DM activity [131]. Thus far, no unbound DO has been found in cells; rather, it associates constitutively with DM, and together the complex recycles between the cell surface and late endosomal compartments [132,133]. It was first suggested that DO inhibits DM, as MHCII–CLIP complexes accumulate at the cell surface when DO is also expressed [134]. After the first DO loss-of-function murine model it was noted that the inhibition of DM activity by DO could be pH-dependent [135]. Interestingly, Kropshofer *et al*. [136] reported that DO could co-chaperone DM and positively influence peptide exchange *in vitro*. Poluektov *et al*. [137] reported a similar effect, and recently acidic pre-treatment of DM or DM–DO was shown to modulate peptide-exchange activity of DM [138]. However, to date, it is assumed that DO affects antigen presentation of peptides that are bound to MHCII proteins under mildly acidic conditions [135]. DO's presence would impede DM function in endosomal compartments, and would act to alter the peptidome presented by MHCII molecules, as kinetically stable peptides cannot be efficiently selected for [139]. Fallas *et al*. [140] showed that ectopic expression of DO in murine DCs and subsequent antigen presentation assays results in antigen-specific down-modulation of class II processing and presentation. Kremer *et al.* [141] have demonstrated that DO inhibition results in the specific activation of CD4^+^ T cells recognizing self-antigenic peptides of relevance for cancer immunity. Interestingly, DM interacts with DR and DO via the same molecular interface, and many of the key residues at this interface are conserved [84,86]. The interaction of DM–DO, however, is considerably stronger than that of DM–DR, as the measured FRET efficiencies are considerably higher in a cellular context [115]. How the interaction between DM and DO is affected by natural polymorphisms in DM, or even in DO, is not yet known at the cellular level; likewise, the effects of these differences, if any, on DM's cellular function remain uninvestigated.

### 2.4. The impact of HLA-DM activity at the organism level: T-cell development, T-cell responses and autoimmune models of disease

The activity of DM at the organism level was addressed for the first time in 1996 by three different groups [142–144] and extended to a number of MHCII backgrounds, as summarized in table 2. These initial studies focused on the targeted deletion of the *H2-Ma* gene in a particular murine model bearing the MHCII haplotype H-2^b^. In essence, the three studies reached the same conclusions: lack of DM results in normal expression of the MHCII molecules that then, in turn, accumulate at the cell surface loaded mostly with the placeholder peptide CLIP. However, conformation-specific antibodies revealed slight structural differences in the nature of the complexes found in two of these three reports [143,144]. Moreover, H2-DM^−/–^ APCs were unable to stimulate restricted hybridomas *ex vivo* during antigen presentation assays, suggesting that antigen processing was impaired. The dysfunction of antigen processing observed in the three studies revealed that thymic selection during T-cell development was altered at two different levels. First, positive selection was partially impaired, and the total number of CD4^+^ T cells was reduced by around 30–50%. Second, negative selection was severely affected in all of these cases, and CD4^+^ T cells selected in the KO mice were unresponsive to H2-DM^−/–^ APCs, but hyper-reactive to wild-type syngeneic cells. Thus, although none of the models displayed a clear autoimmune phenotype, the release of self-reactive CD4^+^ T cells into the periphery was evident. It is important to note that the H-2^b^ haplotype, and in particular the *I-A^b^* allelic variant expressed in these murine models, possesses a high affinity for CLIP [145]. The authors of these studies concluded that both positive and negative selection processes were primarily driven by a single peptide–MHCII complex, CLIP-I-A^b^. Under these conditions, in an MHC background of high affinity for CLIP, DM is essential for CLIP dissociation, allowing MHCII loading with a broader peptidome. Such a broadened peptidome is, in fact, a requirement for efficient negative selection.

**Table 2.** Summary of H2-M^−/–^ murine models and phenotypes. CLIP affinity values have been obtained from [145,146]. Floppy dimers refer to the appearance of the SDS-stable dimers, in this case not running as a compact species. Epitope lost refers to the fact that staining in flow cytometry experiments with monoclonal antibodies is lost in the case of DM KO murine models. n.d. stands for not determined in the same study, thus not directly comparable.

haplotype	MHCII restriction (CLIP affinity, nM)	SDS-stable dimers/CLIP accumulation/MHCII expression	CD4^+^ T cells positive selection negative selection	T-cell hybridoma stimulation	refs
H-2^b^	I-A^b^ (74)	yes (floppy) yes no	reduced 50% strong reactivity towards congenic	impaired	[142–144]
	I-E^b^ (1180)	**—**			
H-2^d^	I-A^d^ (5.3)	no yes no	reduced 50% peripheral expansion efficient	partially impaired	[147,148]
	I-E^d^ (1180)	no yes epitopes lost			
H-2^k^	I-A^k^ (13333)	no yes	near to normal (not shown) sufficient for congenic strains	change in immunodominance	[149]
	I-E^k^ (304)	yes yes			
H-2^g7^	I-A^g7^ (n.d.)	no no yes	reduced increases T_reg_	not tested (I-A^g7^ molecules are able to bind peptides)	[15]

The consequences of DM inactivation, leading to the formation of a single peptide–MHCII complex (CLIP-H2-A^b^) that prevents negative selection but still drives positive selection to a certain extent, were further investigated in a model expressing a defined haplotype including a transgene (A^b^+Ea^k^) [150], another model bearing a single MHCII-peptide complex (A^b^EpIi^−/–^) [151], and in other transgenic models [152]. These studies demonstrated the need for a broad spectrum of self-antigenic peptides during positive selection in order to generate a complete and functional TCR repertoire. The consequences of DM loss in mixed haplotypes (combining I-A and I-E MHCII allotypes) were studied in more detail leading to the conclusion that for those MHCII alleles with low affinity for CLIP (I-Ab combined with I-E^k/b^ or I-A^k^), positive selection was less affected. These studies allowed researchers to propose that the intrinsic ability of certain MHCII allotypes to be loaded with a broader spectrum of peptides in a DM-independent manner may allow for the generation of the self-peptidome required for functional positive selection [153]. Similarly, studies using strains bearing other MHCII alleles with low affinity for CLIP (H-2^d^ haplotype: I-A^d^-I-E^d^) [147], the absence of Ii [154] or even the presence of Ii mutated to decrease its affinity towards MHCII alleles (in a haplotype with high CLIP affinity, H2-A^b^) [155], reached the same conclusions. Conversely, DM inactivation in a haplotype with low CLIP affinity (H-2^k^: I-A^k^-I-E^k^) [149] demonstrated that the relaxed requirement for peptide exchange of the MHCII proteins expressed by this model allowed effective negative selection, and there was essentially no reactivity of purified CD4^+^ T cells from these KO mice in antigen presentation assays with nearly congenic irradiated splenic cells. However, in contrast to the H-2^b^ haplotype, there were lower expression levels of MHCII proteins in the mutant mice, which also showed structural differences.

Recently, another study has also addressed T-cell development upon DM targeting another MHCII haplotype that is directly related to T1D (H-2A^g7^; bearing only functional I-A^g7^ MHCII, which has a very low affinity for CLIP): the non-obese diabetogenic (NOD) mouse. This study showed a clear decrease in the numbers of CD4^+^ T cells, as well as lowered MHCII expression levels and structural differences between the MHCII molecules expressed in the *wt* NOD model versus the DM^−/–^ mutant [15]. More importantly, for the first time DM function was directly linked to an autoimmune disorder at the organism level, and, surprisingly, the absence of DM protected the animals from development of T1D. Besides these studies, Rajagopalan *et al.* [156] created two humanized MHCII (DQ8 and DR3) DM^−/–^ mouse models and analysed T-cell development and certain features of antigen presentation. Essentially, both models lacking DM showed normal T-cell development, and the expression of MHCII proteins was not affected. However, the DQ8 model lacking DM showed a compromised ability to present antigens.

To date, only a few studies at the organism level have investigated how the absence of DM or its modulation by DO affect T_reg_ development. A first study used a ‘limited’ (LTD) model with a restricted TCR repertoire crossed with H2-M^−/–^ H-2^b^ to determine how the TCR usage was changed in T_reg_ versus conventional T cells (including CD4^+^ and CD8^+^ populations) upon DM inactivation [157]. Differences in the TCRBV usage were determined and grouped, and it was found that TCRBV usage differed in both conventional T cells and T_regs_, and was also altered upon DM deletion. However, preferential TCRBV usage under each condition was not attributed to any particular TCR specificity. The recent study by Morgan *et al*. [15] showed higher numbers of T_regs_ in DM^−/–^ mice, which were hypothesized to protect animals from disease. In the study by Yi *et al*. [14] in which DO was constitutively expressed in DCs—and DM activity is therefore reduced—of the NOD mouse model, disease protection was also observed, although no apparent difference in T_reg_ population size was noted. This last study highlights the potential for subtle differences in antigen presentation to modulate and prevent autoimmune disease. Indeed, DM activity has also been postulated to be a key regulatory element during T_reg_ development [7,39,158]. Thus, using different MHCII backgrounds and TCR-transgenic mice, it has been demonstrated that T_reg_ development can be induced if an antigen is expressed as self, and that this induction is dependent on the presentation of the antigen [159,160]. Given the same genetic background used by Morgan *et al*. [15] and Yi *et al*. [14], relative amounts of DM seem to have a dramatic impact on T_reg_ development. T_reg_ selection is believed to be based on intermediate affinities [38], and in this regard I-A^g7^ does not require DM activity to exchange CLIP peptides, and could very well be loaded with intracellular peptides [161].

Many of the above-mentioned studies also addressed how the loss of DM function affects CD4^+^ T-cell responses and CD4^+^ T-cell-dependent immune mechanisms. In most cases, the authors reported a lower ability of the H2-DM-deficient APCs to stimulate restricted hybridomas. Additionally, it was also shown that DM focuses and restricts the immune response towards a very limited number of high-stability peptides, which are called ‘immunodominant’ [162]. In the case of the H-2^d^ haplotype, which combines allotypes with a very high and a very low affinity for CLIP (I-A^d^ and I-E^d^ MHCII proteins), The Bikoff lab [148] determined that for H2-DM^−/–^ mice, not only is the immunodominance of antigenic peptides broader when DM is absent, but the entire immune response shifts from I-A^d^ presentation in the wild-type to I-E^d^ presentation in the KO mouse. This shift in the immune response was at least partially attributed to a higher number of I-E^d^-restricted CD4^+^ T cells in the periphery. A unifying conclusion from all of these studies is that in the haplotypes bearing MHCII allotypes with a low affinity for CLIP, the antigen-presenting function seems to be less affected. Similar to the dependency on Ii for antigen presentation of some epitopes [163], DM dependency could be established and confirmed for some antigens [67]. In the context of autoimmunity, it has been also demonstrated that DM function is essential for the presentation of epitopes involved in the pathogenesis of experimental autoimmune encephalitis (which is a model of human MS) [164].

Tourne *et al.* [150] also described that DM^−/–^ mice were able to respond to certain viral infections, although antibody responses were generally less efficient than in the wild type mouse. A recent paper from the Eisenlohr laboratory has demonstrated that only some viral epitopes require the presence of DM for presentation, and that efficient immune responses rely mostly on the antigen processing of endogenously synthesized virions [165]. In the case of bacterial infection, however, DM seems to be required for an appropriate immune response [166]. The requirement of DM function for mounting efficient antibody responses to pathogens was later evaluated, and the absence of DM was associated with an impaired ability to form germinal centres for B-cell maturation [167].

## 3. HLA-DM and disease studies

In the above sections we have brought the function of HLA-DM into the context of its role as peptidome editor for MHCII proteins. We have also highlighted the importance of DM for the proper function of antigen presentation during the adaptive immune response, as well as during T-cell development. Its central role in antigen presentation, as well as loss of function observed in KO murine models, has motivated a number of genetic association studies aimed at establishing a connection between DM polymorphisms and immune disease. We propose that under conditions of predisposition to disease, where alleles conferring risk to autoimmunity are expressed, DM activity determines whether pathogenic peptides are presented or not, and to what extent this presentation takes place. Under these conditions, a partial loss of function due to inefficient expression, translation, turnover and/or dysfunctional enzymatic activity of DM could represent secondary links to autoimmunity, which would be difficult to address by genetic studies. Immune disorders related to the expression of MHC genes have been found and have been linked to deficiencies of CIITA and RXF. This heterogeneous group of diseases, known as BLS, is characterized by a lack of constitutive, as well as inducible, MHC expression. As may be expected, the major consequence of this lack of MHCII expression is a severely impaired response to pathogens (reviewed in [80,168]). To the best of our knowledge, a transcription or translation deficiency specific for DM due to its promoter or coding regions has yet to be described. Downregulation of DM activity, however, has been detected in patients suffering RA, although this is unrelated to polymorphisms in their promoters [81]. Modulation of DM activity by DO could be another mechanism leading to variable activity levels of cellular DM that may have an impact on autoimmunity. This mechanism operates mostly on B cells and some thymic epithelial cells, although it seems to also be important in other APCs, and is a possibility that has been recently considered [158]. Finally, catalytic impairment of DM proteins has, until only recently, been an unexplored option to explain variable DM activity. DM heterodimers containing a particular allele, DMA*0103, have been shown to be less effective in catalysing the peptide-exchange reaction compared to the most abundant allele DMA*0101 [12]. This work has demonstrated that catalytic impairment of DM is an unexplored, but attractive, possible mechanism that may influence autoimmune conditioning. The complexity of the peptide-exchange reaction together with the above-mentioned inconclusive genetic studies reveal that much remains to be understood about antigen processing and presentation with regard to autoimmunity.

### 3.1. Genetic association studies of DM and immune disorders

While a clear link between classical MHCII polymorphisms and disease exists, genetic association studies connecting HLA-DM variants and autoimmunity remain controversial. To date, several genetic investigations were unable to show unequivocally that HLA-DM polymorphisms represent an additional risk factor in RA or T1D (table 3). Although these studies are methodologically sound, there are several important factors to consider regarding their interpretation. Most importantly, the impact of HLA-DM activity will depend not only on the particular *DMA* and *DMB* alleles under investigation but also on the combination of MHCII alleles expressed by an individual. It is therefore necessary to know the genotype of both patients and controls, in order to first determine the risk of disease, and then to observe whether certain polymorphic variants contribute to the onset of the disease. DM is a heterodimeric protein whose two subunits are involved in the interaction with MHCII molecules, and are therefore also involved in the catalytic mechanism. Hence, the activity of DM as an enzyme would depend on the particular pairing of *DMA* and *DMB*. Therefore, studies that fail to address these issues and exclude one of the genes encoding any of the subunits may need to be reinterpreted in light of the current knowledge. In line with our view, Sirota *et al*. [180], combining their own dataset with independent studies, were able to demonstrate that among 563 SNPs, the *DMB* SNP rs151719 (intron) was linked to a number of autoimmune disorders with opposite risk profiles. In the case of RA and T1D, this SNP (rs151719, in an intron) results in protection, while in the case of MS this SNP is considered to confer risk. Moreover, the authors speculated that this specific feature of opposing risk profiles may result from differences in the pathogenesis of each disease.

**Table 3.** Genetic association studies of HLA-DM natural variants with autoimmune disorders.

disease	MHCII	association	DM association LD to MHCII (classical)	ref.
rheumatoid arthritis	DRA1*0101 + DRB1*0101 or DRB1*0401	positive	DMB alleles 1 time each —	[169]
		positive	DMA*0103 only when combined to DR1 No LD	[170]
		positive	DMA*0101 No LD with DR	[171]
		negative	n.s. DMA*0102/*0104 decreased frequencies in patients n.s. LD	[172]
		positive	DMB*0101 to any DR n.s. LD	[173]
		positive	DMA*0103 to any DR n.s. LD	[174]
		negative	— LD DMB*0103 and DRB1*0101 and *0401	[175]
		negative	— —	[176]
type 1 diabetes	DRA1*0101 DRB1*0401 or DQA1*0301 DQB1*0302	positive/protective	DMA*0101/DMA*0102 No LD	[177]
		positive (LD)	DMB*0102 and DMB*0104 LD DRB1*04 or DRB1*0302	[178]
		negative	— no LD	[179]

Recently, Feng *et al*. [181] found certain *DMA–DMB* combinations to be more frequent in the Han Chinese population, which could be interpreted as different *DMA*–*DMB* haplotypes. However, the same authors indicate that *DMB* frequencies in other populations vary significantly, and that this should then lead to different DM haplotype frequencies. It is also important to note the striking differences between the LD observed in these studies with regard to DM genes and classical MHCII molecules. There is a recombination hotspot between *DMB* and *TAP2* that has been proposed to make an association between *DR-DQ* alleles and DM haplotypes unlikely (figure 1*a*, shown as a red cross) [72]. Indeed, among these studies, only one found a clear LD between *DMB-DQ* and *DMB-DR* [178]. The strong LD between DMB*0104 and DQB1*0201 reported was argued to exclude a direct association between DM and T1D. Thus, the clear association of the two *DMB* polymorphisms reported was considered a secondary link to T1D. All of these considerations show the inherent difficulty in fully deconvoluting the causative aspects of autoimmune disorders such as RA and T1D on a purely genetic basis. However, a recent study has reported a significant correlation of SNPs resulting in the DMA-V142A mutation found in DMA*0102 with hepatitis C virus infection and clearance [182]. Additionally, an intronic SNP variant has recently been linked with susceptibility to HIV-mediated Kaposi's sarcoma [183].

### 3.2. HLA-DM's contribution to autoimmune disorders

Although researchers have found common mechanisms acting during the development of autoimmunity [1], the aetiology and immunopathogenesis seem to be different for each disorder. In this context, when approaching any contribution DM may make towards increased risk of disease, it is necessary to understand and appropriately delineate what role MHCII antigen presentation plays in the development of the disorder, and what primary cell types are involved. Two clear examples of MHCII-mediated autoimmunity for which there is a thorough and well-documented understanding of the immune effector mechanisms implicated in disease are T1D and RA. These two disorders seem to have both a humoral and a cellular component, and recent studies provide evidence for a role of DM in the onset of these diseases. Additionally, an interesting and common feature of many MHCII molecules mediating autoimmunity is a low affinity for CLIP [16]. Examples of interest that share this signature low affinity for CLIP include DQ8 [184–186] and DR4 [117].

#### 3.2.1. Rheumatoid arthritis

RA is characterized by the inflammation of the synovial lining of joints, which ultimately leads to cartilage damage. The contribution of the cellular component of the immune system is evident, as passive transfer of autoreactive CD4^+^ T cells is also able to trigger the disease [187]. Additionally, HLA-DR1 and -DR4 have been shown to be the most prominent risk alleles for the disease. In RA, most studies to date have focused on the presentation of peptides from CII, which is the major component of the connective tissue [188]. In 1996, Walter *et al*. [78] studied the distribution of H2-DM polymorphisms in mice strains susceptible and non-susceptible to collagen-induced arthritis (the experimental mouse model for RA). Murine *H2-DM* differs from that of humans, as there is a duplication of the *H2-Mb* gene leading to *H2-DMb*1 and *H2-DMb*2, as described above. Interestingly, it was found that certain *H2-DMa* and *H2-DMb* alleles are present only in disease-susceptible strains [78]. In 2008, Amria *et al*. [122] used MHCII-negative B-cell and macrophage lines to assess the impact of DM and Ii on RA. In this case, the authors were able to provide evidence that the DR4-restricted CII immunodominant epitope was less efficiently presented in the presence of DM; additionally, hybridoma T cells specific for this epitope proliferated to a significantly lower extent in the presence of DM. The use of different drugs inhibiting cellular uptake of the antigen or intracellular trafficking processes allowed the authors to determine that DM activity was necessary to eliminate the CII peptide that binds to DR4, and that CII peptide release of CII–DR4 complexes takes place in recycling endosomes. In line with these observations, Louis-Plence *et al*. [81] showed lowered levels of DM in APCs of patients suffering from RA, and they could demonstrate that this is not related to DM's promoter activity. Hence, low DM protein levels seem to be related to the onset of arthritis. Additionally, anti-citrullinated protein antibodies (ACPAs) are early indicators of RA and are detectable even before diagnosis. Those autoantibodies are secreted by B cells [189] in which DM activity plays an essential role for affinity maturation and is modulated by DO expression [167]. There are currently several hypotheses explaining how HLA genes and ACPAs connect to disease, and it seems that the contribution of specific CD4^+^ T cells is essential [190]. It is therefore not unlikely that mechanisms involved in antigen presentation could also contribute to the activation of such specific CD4^+^ T cells.

#### 3.2.2. Type 1 diabetes

The role of H2-DM in the onset of T1D has been recently addressed using a murine model [15]. T1D is a metabolic disease resulting from the immunologically mediated destruction of the pancreatic beta cells of the islets of Langerhans. The contribution from MHC accounts for around 40–50% of the inherited disease risk. Autoreactive CD8^+^ T cells, as well as CD4^+^ T cells, contribute to disease development, as MHC-presented insulin fragments are one of the most important targets for both T-cell types [191]. The most relevant APC for self-reactive CD4^+^ T-cell activation in this case are DCs [192,193]. Additionally, B cells and autoantibodies directed towards self-antigens also recognized by T cells seem to play also a role in the immunopathogenesis of T1D [194]. The knowledge is more limited than for RA and is restricted to a few murine models; however, it has been shown that B cells expressing surface but not secreted IgM develop a pathogenic phenotype [195]. Similarly, B cells bearing a B-cell receptor repertoire biased towards islet antigens result in rapid development of disease [196] and, of particular interest for this review, the deletion of MHCII genes in B cells results in protection from T1D [197].

Morgan *et al*. [15], by performing a knockout of the *H2-DMa* gene in the NOD mouse background, demonstrated that the presence of DM was required for T1D development. Interestingly, deletion of DM in NOD mice expressing exclusively I-A^g7^ (an analogue of HLA-DQ8 in humans) also resulted in complete protection from disease. I-A^g7^ interacts normally with DM [198] and binds CLIP with a relatively low affinity [16]. However, and most important for disease, the binding properties of I-A^g7^ and certain DQ alleles seem to be unique [199]. Most of the T1D-predisposing alleles, including DQ2 and DQ8 in humans, share non-DQβAsp57 residues in the P9 pocket. The low-affinity binding of self-antigenic peptides during thymic selection allows autoreactive CD4^+^ T cells to escape tolerance mechanisms. However, elevated presentation of insulin-derived peptides on DCs in the islets of Langerhans that have taken up insulin granules of beta cells increases the likelihood of T-cell responses towards those complexes [13]. The main features of the *H2-DMA* KO model were lower cellular surface expression of I-A^g7^ and defective occupancy of these molecules, but the animals were nevertheless protected from the disease. Increased numbers of CD4^+^CD25^+^ T_regs_, but not of pathogenic CD4^+^ T cells, were also observed. These results highlight the role of HLA-DM in both negative and positive selection mechanisms, not only for CD4^+^ T helper cells but also for CD4^+^CD25^+^ T_reg_ cells [15]. Other studies have also correlated DM activity to T1D, with observations that shifting of the insulin-binding register to I-A^g7^ [13] and presentation of the immunodominant epitope of GAD65 by HLA-DR4 [121] are both strongly influenced by DM activity. Similar to the results of the study by Morgan *et al*. [15], constitutive expression of DO in DCs prevents the onset of T1D from very early stages in the same NOD I-A^g7^ murine model [14,140]. Thus, the studies by Morgan *et al*. [15] and Yi *et al*. [14] provide direct evidence for the implication of DM activity levels in T1D. A recent review by Denzin [158] emphasized the role of DO as a modulator of DM levels in B cells, highlighting the importance of DM activity in the development of T1D. Additionally, Stern & Mellins [17] have also reviewed the influence of DM function on T1D, indicating that while the studies by Morgan *et al*. [15] and Yi *et al*. [14] support a role of DM in disease initiation, the studies by Mohan *et al*. [126] and Stadinski *et al*. [127] indicate a contribution of DM for disease pathogenesis.

## 4. Hypothetical contribution of DM to autoimmune disorders

Our recent investigations suggest that polymorphisms of HLA-DM-encoding genes could result in HLA-DM variants with differential enzymatic activities. The most obvious effect polymorphisms may have on DM would be on its editing and/or chaperone functions. We assume that these differences in HLA-DM function would result in changes in the overall population of MHCII proteins and in the population of peptides presented by MHCII molecules (figure 3). There might be important differences in the catalytic behaviour of DM allotypes *in vitro* and in a cellular context. Thus, we propose that the biochemical observations we have recently described [12] have to first be translated into a cellular context before further functional conclusions are drawn. The biochemical features of DM and DR heterodimers are well correlated to their behaviour in cells; however, the catalytic activity of DM *in vitro* in the presence of the transmembrane domains is about 400-fold higher when compared with the activity of the proteins bearing only the ectodomains [200]. The twofold difference in peptide exchange activity detected by us for the DMA*0103 heterodimers *in vitro* could be more pronounced for HLA-DM functions *in vivo*, especially when amplified by additional constraints in the cell, such as membrane anchorage. We therefore propose that DM polymorphisms could differentially affect DM cellular functions, with major consequences at the peptidome level and in the amount of functional MHCII proteins available for antigen presentation.

**Figure 4.**
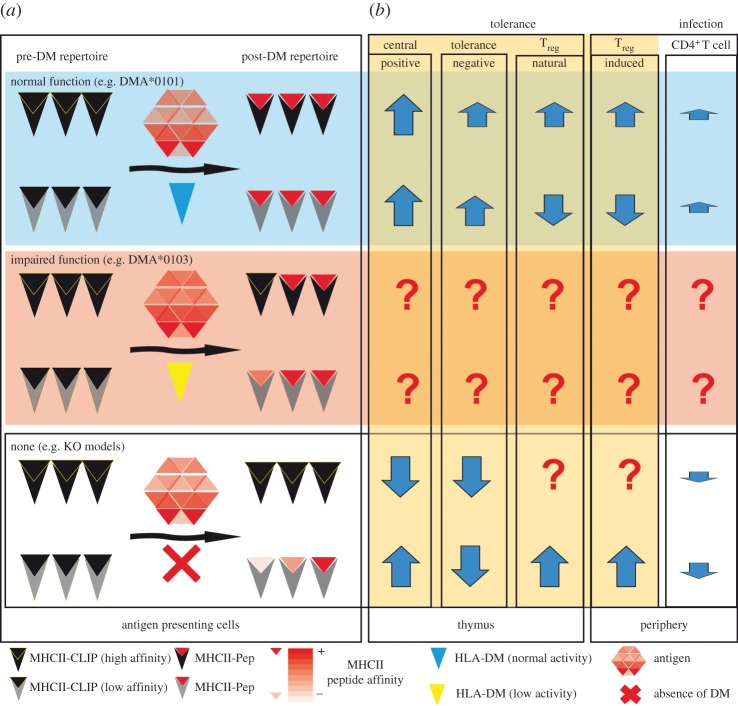
Expected impact of low DM editing activity on the MHCII-associated peptidome and its consequences for tolerance to self and peripheral presentation of self- and foreign antigens. (*a*) Overview of how HLA-DM affects the peptidomes presented by MHCII proteins with high affinity (black ‘V's) or low affinity (grey ‘V's) for CLIP (small black inverted triangles) in the presence of normal (upper panel, large blue inverted triangle), catalytically impaired/low (intermediate, large yellow inverted triangle) or no DM (red ‘X’) activities. Low DM activity (large yellow inverted triangle) could represent defects at transcriptional, catalytic or modulation levels. Antigenic sources contain a number of potential binding epitopes with different affinities for the particular MHCII. DM function favours the binding of high-affinity epitopes. The arrows represent the relevant antigen processing conditions for each antigen (unfolding and/or proteolysis). (*b*) The MHCII peptidome impacts a number of different immune processes related to T-cell development, tolerance acquisition and adaptive immune responses to pathogens. For some of the different DM editing conditions described in (*a*), it has been shown how DM loss affects most of these processes for both high and low MHCII affinities for CLIP alleles. T-cell numbers with respect to the normal DM editing levels are reduced when the arrows point up or increased when they point down. Although there are expected changes in the peptidome presented by MHCII proteins, it is difficult to predict the consequences in terms of T-cell numbers.

CD4^+^ T cells sample the MHCII-peptidome via their TCR and, upon recognition of specific peptide–MHCII complexes, initiate signalling events, ultimately resulting in either the activation or the apoptosis of the particular CD4^+^ T cell. The MHCII-associated peptidome has been demonstrated to be affected by the presence and the expression levels of HLA-DM, among other factors. Therefore, enhanced or decreased presentation of particular peptides within the MHCII-associated peptidome might be affected by DM allotypes with differential catalytic activities (figure 4*a*). Important processes where changes in the MHCII-associated peptidome could potentially be affected by differential DM catalytic properties include central tolerance mechanisms, T_reg_ development, and differentiation and peripheral antigen presentation under both steady-state conditions and in response to pathogens (figure 4*b*). Therefore, DM allotypes with different catalytic properties could lead to significant differences in the displayed peptidome in any of the above-mentioned processes. Such differences at the peptidome level would then substantially contribute to disease conditioning.

It is important to note that not all MHCII proteins have the same requirement for active DM to catalyse peptide exchange. Thus, most biochemical and cellular investigations have focused on understanding how DM activity impacts restricted antigen presentation. It is generally accepted that there are differences between MHCII alleles with low and high affinity for CLIP. Thus, in the case of MHCII alleles with high affinity for CLIP (figure 4*a*, black ‘V’), the absence of DM activity results in the accumulation of CLIP in the MHCII-associated peptidome. Conversely, in the case of MHCII alleles with low CLIP affinity (figure 4*a*, grey ‘V’), even in the absence of DM activity, high-affinity or high-abundance peptides can replace CLIP and be transported to the cell surface. In the presence of DM, however, the MHCII-associated peptidome is biased towards high-affinity peptides, which are selected mostly in MIIC compartments. In the case of DM allotypes with reduced catalytic activity (red background, e.g. DMA*0103), it is likely that an intermediate situation between normal DM function allotypes (blue background, e.g. DMA*0101) and total loss of function (white box) exists. In the presence of DM allotypes with low catalytic activity, the MHCII-associated peptidome will likely include high-affinity peptides (for MHCII alleles with both low and high affinity for CLIP). However, we hypothesize that there will be differences in the composition of the peptidome representing intermediate-to-low-affinity peptides. While in the case of MHCII alleles with high CLIP affinity this epitope could still be represented in the peptidome, for MHCII alleles with low CLIP affinity CLIP will probably be replaced by other antigens.

The consequences of the described changes in the MHCII-displayed peptidome with regard to adaptive immunity and self-reactivity have been investigated for the presence versus the absence of DM in murine models, as reviewed above. Predictions for DM allotypes with low catalytic activities, based on the observed effects in the loss-of-function murine models, might be too simplistic, and the development of more nuanced research models may be required. However, there are a few considerations which are likely to be significant based on the current knowledge of peptide exchange derived from the DM KO models. To date, knowledge of loss of DM function on immunity is mostly restricted to central tolerance mechanisms; two recent reports focus on the consequences of loss of DM function on an allele with low CLIP affinity for the development of T_regs_, and one recent study investigates the TCR repertoire in an LTD DM KO model. According to the murine models reviewed here, it is likely that positive selection will not be substantially affected by allotypes with low DM catalytic activity. However, it is also likely that negative selection mechanisms will be affected, especially in the case of MHCII alleles with high affinity for CLIP. Less efficient release of CLIP from MHCII molecules during negative selection will be likely to result in less efficient binding of TRA-derived epitopes, and thus less efficient deletion of self-reactive CD4^+^ T. In the case of T_reg_ selection in the thymus, it is expected that changes in the peptidome selecting T_regs_ would affect the numbers and specificities of released T_regs_ in a similar manner. The presentation of self-antigenic peptides driving autoimmunity, but also inducing the differentiation of T_regs_, could also be affected by different DM activities. In this case, it is important to note what would be the main APC cell type involved in the presentation of the pathogenic peptides and during the induction of T_regs_. Moreover, induction of T_reg_ differentiation in the periphery would probably be affected as well. Based on the aggregate knowledge represented by the studies cited above, we believe that neither the overall response to pathogen infections would be significantly affected in the case of low DM catalytic activities nor would the antibody response be completely impaired. However, APCs from individuals carrying DM variants with slight but sustained reductions in their catalytic activity could show significant differences at the peptidome level. Such differences could set a biased in the peptidome, ultimately leading to development and/or progression of autoimmune disease.

## 5. Concluding remarks

Many studies have found a direct correlation between dysfunctional DM editing and autoimmune susceptibility. Our recent findings of lower catalytic activity of the DMA*0103 natural variant have opened a new horizon for research in the field of antigen processing and presentation, with particular relevance for autoimmune reactivity. However, to confidently establish a connection between natural HLA-DM variants and disease conditioning, there are two questions that must be addressed. First, to what extent are our *in vitro* results translated into a proper cellular model of DM function, and, second to what extent do DM variants result in different levels of CD4^+^ T-cell activation? Although DM has an enzyme-like behaviour, it is essential to keep in mind that, in order to properly address this question, a system must be designed with as few differences in the natural DM/MHCII expression ratios as possible. Only then could one properly estimate the impact of natural substitutions in an unbiased context. The second relevant question is how *DMA* and *DMB* genes associate in human populations. To date there is a paucity of information on how *DMA*–*DMB* genes associate, and it is likely that the level of DM activity will be the direct result of the particular allelic combination that is expressed in a given individual. Indeed, a crucial question arising from the animal models described above is how DM activity would shape the peptidome presented by different MHCII proteins in settings more complex than single-allele *in vitro* assays or monogenetic cell lines. The murine models used thus far clearly demonstrate that the phenotype and consequences of loss of function at the organism level are extremely dependent on the MHCII molecules expressed. Our investigations showed a decreased activity of an allelic variant of DMA (DMA*0103); however, we only focused on HLA-DR alleles. It is therefore important to extend our knowledge first to other classical MHCII proteins and develop biochemical and cellular methods allowing the testing of the observed effects in polygenic settings. The group of Sadegh-Nasseri has developed and applied a reconstituted *in vitro* system to determine immunodominance [201] and preferential processing conditions for self-antigens involved in autoimmunity [202]. This reconstituted *in vitro* system offers an attractive platform for testing the influence of DM in complex with MHCII backgrounds under controlled conditions. On the other hand, the widespread availability of CRISPR-Cas9 genome editing also presents a valuable option for cell line manipulation to study the influence of DM activity at the cellular and organism level.

HLA-DM function is modulated in B cells and some thymic epithelial cells by its interaction with the non-classical MHCII molecule HLA-DO. Because DO engages DM using the same interface that MHCII molecules do, it is therefore not unlikely that this interaction (DM–DO) could also be affected in natural HLA-DM variants. If this is the case, editing capabilities of different DM allotypes in these cells will also be differentially modulated. Whether the DM–DO interaction is affected by natural variations of DM has not yet been systematically investigated, but it remains an important question, as it could also have consequences in central tolerance mechanisms and B-cell activation. Indeed, as already seen after almost 50 years of research on the role of MHCII polymorphisms in autoimmune diseases, it is likely that the contribution, if any, of DM natural variants to disease pathogenesis will apply only to specific mechanisms and not broad, systemic changes. The implications of any HLA-DM natural variant should, however, be considered in the complex context of each particular disease. Because different immunopathogenic mechanisms are assumed to have different implications for each disease, it is important to consider what the role of MHCII polymorphisms are in each case. In the two model autoimmune diseases discussed in this paper, there is a clear connection between MHCII polymorphisms and recognition by CD4^+^ T cells, resulting in autoimmune disease. However, the APC subset of highest relevance for each disease and the impact of DM activity in different processes vary from RA to T1D. Further research on humanized animal models will definitively contribute to our understanding of the role of HLA-DM natural variants in disease conditioning.
